# Low-resolution refinement tools in *REFMAC*5

**DOI:** 10.1107/S090744491105606X

**Published:** 2012-03-16

**Authors:** Robert A. Nicholls, Fei Long, Garib N. Murshudov

**Affiliations:** a Structural Studies Division, MRC Laboratory of Molecular Biology, Cambridge CB2 0QH, England

**Keywords:** low-resolution refinement, *REFMAC*5

## Abstract

Two aspects of low-resolution macromolecular crystal structure analysis are considered: (i) the use of reference structures and structural units for provision of structural prior information and (ii) map sharpening in the presence of noise and the effects of Fourier series termination. The generation of interatomic distance restraints by *ProSMART* and their subsequent application in *REFMAC*5 is described. It is shown that the use of such external structural information can enhance the reliability of derived atomic models and stabilize refinement. The problem of map sharpening is considered as an inverse deblurring problem and is solved using Tikhonov regularizers. It is demonstrated that this type of map sharpening can automatically produce a map with more structural features whilst maintaining connectivity. Tests show that both of these directions are promising, although more work needs to be performed in order to further exploit structural information and to address the problem of reliable electron-density calculation.

## Abstract 2

Low-resolution refinement tools implemented in *REFMAC*5 are described, including the use of external structural restraints, helical restraints and regularized anisotropic map sharpening.

## 1. Introduction

Heterogeneous organization of molecules in the crystal lattice can lead to the diffraction data being of poor quality. Such heterogeneities may arise from effects such as crystal mosaicity, molecule/chain flexibility and localized disorder. This results in weak diffraction intensities, causing the data to be collected using a lower resolution threshold and thus with low information content. This behaviour is often observed for large complexes. However, the structures of the individual components of a complex may have been independently determined at higher resolution. Such information might then be used to aid the refinement of the lower resolution structure.

There are other factors that can reduce the information content of macromolecular crystallographic (MX) data, thus reducing effective resolution. These include crystal-growth peculiarities such as twinning and OD order–disorder. In these cases, although the nominal resolution may be high, not all of the observations are independent. For example, in the case of perfect hemihedral twinning the number of independent observations is decreased by a factor of two, corresponding to a resolution reduction by a factor of 2^1/3^ = 1.26. Therefore, in the limit, the quality of the electron-density map in the presence of perfect hemihedral twinning at 2 Å would correspond to that at 2.52 Å in the single-crystal case. The refinement of models against data from twinned crystals is now routine (Murshudov *et al.*, 2011 ▶; Adams *et al.*, 2010 ▶; Sheldrick, 2008 ▶). However, statistics after refinement against such data should be interpreted with care (Murshudov, 2011 ▶). It is important to remember that *R* factors and other overall statistics are dependent on the statistical properties of the data and therefore comparison of *R* factors from different crystals may give the wrong impression about the comparative quality of the models.

There are many problems that need to be tackled in order to make low-resolution structure analysis routine, two of which are considered in this paper.

(i) The use of chemical and structural information as restraints to increase the consistency of the derived atomic models with available prior knowledge. The use of chemical information in the form of bond lengths, bond angles and torsion angles has always been routine. For details of the organization and use of chemical knowledge in refinement, see, for example, Vagin *et al.* (2004 ▶). Recent years have seen an explosion of approaches towards utilizing structural information (Schröder *et al.*, 2007 ▶, 2010 ▶; Sheldrick, 2008 ▶; Murshudov *et al.*, 2011 ▶; Smart *et al.*, 2012 ▶). This demonstrates the importance of finding a (and the lack of a unique) solution to the problem of exploiting structural information.
(ii) Calculation of electron density to aid in the reduction of errors introduced during manual and automatic model building. Data from low-resolution crystals usually exhibit high isotropic and anisotropic *B* values. This contributes to the observation of smeared regions of electron density, with vanishing side chains, secondary-structural elements and even domains. Were this effect removed, the electron-density map may reveal more features. Current approaches use only one *B* value for crystal map sharpening. However, the problem is complicated by the non-negligible influence of contributing factors such as anisotropic diffraction, rigid-body oscillation of individual structural units and correlated motion of whole chains.

Many tools have been developed to aid crystallographic refinement at medium and higher resolutions over the past few decades. One of the current challenges is to develop complementary approaches for dealing with cases where only low-resolution data are available (lower than around 3 Å). One of the sources of available information is the three-dimensional structures of macromolecules deposited in the Protein Data Bank (Berman *et al.*, 2002 ▶). Structural information may be utilized in various forms, such as secondary-structure restraints, homologous reference structures and homology models, by various modern refinement software packages including *REFMAC*5 (Murshudov *et al.*, 1997 ▶, 2011 ▶) from *CCP*4 (Winn *et al.*, 2011 ▶), *BUSTER-TNT* (Blanc *et al.*, 2004 ▶), *phenix.refine* (Adams *et al.*, 2010 ▶; Afonine *et al.*, 2005 ▶), *SHELX* (Sheldrick, 2008 ▶) and *CNS* (Schröder *et al.*, 2010 ▶; Brünger *et al.*, 1998 ▶).

The concept of calculating an electron-density map showing more features, *e.g.* side chains, has been proposed by many authors. Notably, Brunger and coworkers (Brunger *et al.*, 2009 ▶; DeLaBarre & Brunger, 2006 ▶) have suggested a procedure known in the field of image processing (Gonzalez & Woods, 2002 ▶) as inverse filtering. However, it is known that such filters can amplify noise, thus masking out real signal. Unfortunately, the electron density always contains noise, which stems from several sources.

(i) Noise arising from variations in the experimental data.
(ii) Noise arising from errors in the model (*e.g.* atomic coordinates, model incompleteness, misparameterization, *B* factors, scale factors) and thus in calculated phases. Such noise correlates with the ‘true’ electron density and is consequently very hard to address.
(iii) Noise arising from Fourier series termination. When data are collected to the crystal diffraction limit and no map sharpening is used, such noise usually dies out approaching the high-resolution limit. However, when map sharpening is used as an inverse filter then the effect of series termination becomes pronounced.

In this paper, we first describe the use of external structural information, specifically inter­atomic distance restraints obtained from reference homologous structures and general fragment-based restraints (including quasi-secondary-structure restraints, in particular to helical conformations). We then describe anisotropic regularized map sharpening. For each method, we provide examples of usage.

## 2. Structural restraints

### 2.1. Application of external structural restraints in crystallographic refinement

Information from external sources can be incorporated during refinement using a Bayesian framework where distribution of interatomic distances serves as prior knowledge. Thus, restraints generated using external structural information should help the macromolecule under refinement to adopt a conformation that is more consistent with previous observations. This is similar to the use of geometry terms in refinement, which helps local structure adopt chemically reasonable conformations.

The minus log posterior distribution target used in *REFMAC*5 (Murshudov *et al.*, 1997 ▶) may be expressed 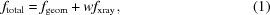
where *f*_geom_ and *f*_xray_ are the contributions of geometry terms (−prior distribution) and experimental data (−log likelihood) and *w* weights their relative contributions. The geometry component is a linear combination of various factors (effectively equivalent to the assumption that these contributors are independent), including any available external structural information,
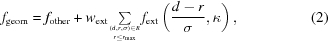
where *f*_ext_(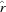, κ) is the unweighted contribution of an external interatomic distance restraint (*d*, *r*, σ) ∈ *R* to the target function, where *R* is the list of external restraints and the function *f*_ext_(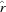, κ) depends on the normalized residual 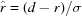 and parameter κ. The parameter *w*_ext_ adjusts the weight of the external restraints relative to the other geometry components and *f*_other_ represents the contribution of all other prior information (Murshudov *et al.*, 2011 ▶). An interatomic distance restraint comprises the current distance *d* between two atomic positions, the objective value *r* and the standard uncertainty σ. The mechanism used for application of external restraints in *REFMAC*5 is described by Mooij *et al.* (2009 ▶).

Here, we stipulate that the objective value *r* of an external restraint should be lower than some threshold *r*_max_, so that only reasonably short-range restraints are utilized, thus allowing resistance to differences in global conformation. In *REFMAC*5, the Geman–McClure robust estimation function (Geman & McClure, 1987 ▶) is used for external restraints,
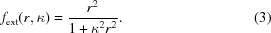
This function, which is equivalent to least squares for κ = 0, helps to reduce the influence of outliers and sensitivity to conformational changes.

Various criteria have been used for optimization of the X-­ray weight *w*, notably *R*_free_ (Brünger, 1997 ▶) and −LL_free_ (Bricogne, 1997 ▶; Tickle, 2007 ▶). Similarly, the appropriate selection of the external weight *w*_ext_ is not obvious. One potential optimization criterion might be to minimize *R*_free_. However, it should be acknowledged that this may reduce the usefulness of *R*_free_ as an independent indicator of refinement quality. Therefore, the weight for external structural information *w*_ext_ requires careful consideration.

#### 2.1.1. Selection of external structural information

External structures should be selected on the basis of their reliability and similarity to the current model. For example, suitable reference structures may include sequence-identical, homologous or structurally similar models solved at a higher resolution or generically derived structural information from nonhomologous sources (*e.g.* secondary-structure restraints obtained from an ideal α-helix).

The use of external restraints may in some cases be justified by any resultant increase in the reliability of atomic positions. However, it should be acknowledged that such an approach introduces bias; the influence of such bias may result in the model adopting a conformation that is less consistent with the observed data. The use of external restraints might make a particular model adopt a conformation very similar to a high-resolution homologue, assuming it is appropriate to do so, and ideally result in improved refinement statistics.

We suggest that external restraints should only be used if the benefits of any improvement in reliability are deemed to outweigh the negative effects. Indeed, this may well be the case for data of poor quality collected at low resolution. For example, refinement of a model might cause some regions of very poor electron density to adopt an incorrect conformation. Increasing the weight of geometry terms may help the structure to adopt a more chemically reasonable configuration, but the region may still be incorrectly modelled owing to the effect of the misleading density; geometry restraints operate at a very high level of structural resolution. However, external restraints can operate at a lower level of structural resolution, as desired (by increasing the maximum restraint distance *r*_max_; see below).

### 2.2. External restraint generation

Here, we shall refer to the chain that is to be refined as the *target* chain and to the chain that is to be used to generate the restraints as the *external* or *reference* chain.

External restraints for use in refinement by *REFMAC*5 may be generated using the *ProSMART* tool developed by Nicholls (2011 ▶). Amongst various other functionalities, *ProSMART* can generate restraints on interatomic distances utilizing structural information. Whilst reference structures would generally be near-identical in sequence, the approach allows the alignment of, and subsequent restraint generation using, any reference chain(s). However, it is not implied that there would be any utility in using external restraints based on dissimilar structures; a high degree of local structural conservation would generally be required for the successful application of external restraints. In general, we assume that the target and external reference structures are sufficiently similar, although such decisions should ultimately be made by the user.

The alignment approach adopted by *ProSMART* is independent of global conformation, instead being concerned with the net conservation of local structure at a chosen level of structural resolution. Indeed, the restraints generated by *Pro­SMART* allow great global flexibility rather than rigidly pulling the target structure towards the same global conformation. Consequently, it is not necessary for the external reference chain to adopt the same global conformation as the target, *e.g.* structures in different bound states can be used. It is, however, necessary for local structure to be sufficiently well conserved along the chain so that the effect on refinement is positive.

#### 2.2.1. General approach

Suppose that we want to generate an interatomic distance restraint between two atoms in the target structure given knowledge of their positions and thus the distance *d* between them. Given an external reference structure and a residue alignment between the target and reference structures, it is possible to find the distance *r* between the corresponding atoms in the reference structure. The distance *r* is the objective value of the restraint.

If the target and external chains share a high degree of structural similarity, then we might expect *d* to be approximately equal to *r*, with some error. Consequently, the restraint distances *r*, with appropriate distributional assumptions, can be used as prior information during crystallographic refinement. Since we want to maintain a degree of global conformational independence between the target and reference structures, it is undesirable to generate restraints between atoms that are far apart. Therefore, restraints are only generated whose objective values are less than some threshold *r*_max_. This parameter represents the structural resolution of the restraints; lower thresholds allow better conformational independence, whilst higher thresholds provide more information about the surrounding structural environment.

The adopted procedure of external restraint generation thus involves the identification of lists of corresponding intrachain atom pairs in both the target and reference structures, filtering these lists in order to identify only those atom pairs suitable for restraint generation (based on interatomic distance criteria), the identification of corresponding atom pairs between the target and reference structures and finally estimation of restraint distributions.

#### 2.2.2. Identification of atom pairs to be restrained

Knowledge of an alignment between residues allows the direct inference of an atomic correspondence between target and reference structures. Such a correspondence may include both main-chain and side-chain atoms (providing the aligned amino acids are the same) or only main-chain N, C^α^, C and O atoms (allowing main-chain structural restraints to be generated even for residues of different amino-acid type). The alignment may also be filtered according to conservation of local main-chain and/or side-chain structure in an attempt to avoid the generation of potentially unsuitable restraints.

Given an alignment of atoms, it is then necessary to identify a list of sufficiently close atom pairs independently for each of the two structures. Various methods of near-neighbour searching have been developed. Here, in order to efficiently reduce the search space, we use a cell technique (Bentley, 1975 ▶) previously used in biology (Levinthal, 1966 ▶), which involves the uniform partitioning (voxelization) of space into cubic cells with edge length *r*_max_ (the value of *r*_max_ is chosen to be 1.5 times greater for the target structure than for the reference). This approach allows the efficient identification of all atoms with positions *x_i_* and *x_j_* such that their interatomic distance satisfies the criteron *r*_min_ ≤ |*x_i_* − *x_j_*| ≤ *r*_max_.

Using the achieved atomic correspondence, we may then calculate the list of all pairs of corresponding atom pairs, only considering those identified as being sufficiently close. The quantities of interest directly follow, namely the interatomic distance *d_ij_* = |*x_i_* ^target^ − *x_j_* ^target^| between atoms *i* and *j* in the target structure and the distance *r_ij_* = |*x_i_* ^ref^ − *x_j_* ^ref^| between corresponding atoms in the reference structure.

**Figure 1:**
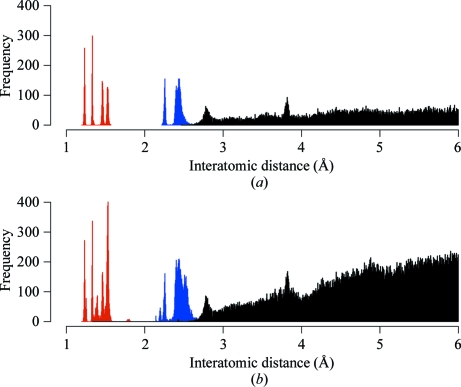
Histograms of the interatomic distances in the structure with PDB code 2jhp (Sutton *et al.*, 2007 ▶) corresponding to (*a*) main-chain atoms only and (*b*) both main-chain and side-chain atoms. Distances corresponding to atom pairs separated by one chemical bond are shown in red, those separated by two bonds are shown in blue and all other atom pairs are shown in black.

Finally, distances between atom pairs that are already tightly restrained by standard geometry terms are removed from the list of external restraints. In particular, we remove any short restraints separated by only one or two chemical bonds (see Figs. 1 ▶ and 2 ▶).

### 2.3. Maximum-likelihood estimation of structural restraint distributions

Removal of distances restrained by standard geometry is vital for estimation of restraint distributions. It is reasonable to assume that the variability of longer range restraints would be very different to that of short restraints separated by only few bonds (see Figs. 1 ▶ and 2 ▶).

#### 2.3.1. Form of the restraint distributions

Suppose the distributions of the positions of two atoms in the target structure are **x**_1_ ≃ *N*(**c**_1_, σ_1_^2^) and **x**_2_ ≃ *N*(**c**_2_, σ_2_^2^), where **c**_*i*_ is the coordinate corresponding to atom *i*. Since we are generally interested in low-resolution structures, we assume spherical normality; the variance terms are scalar to emphasize this point. Note that *B* factors are closely related to the variabilities of these distributions, which are usually chosen to be isotropic for low-resolution structures.

**Figure 2:**
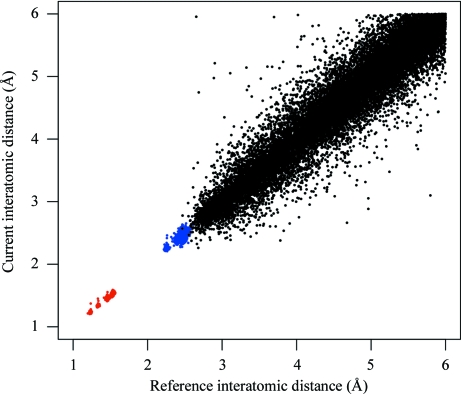
Distance dependence of the distribution of interatomic distances (for main-chain atoms only) for the target structure 2jha (Sutton *et al.*, 2007 ▶) using the sequence-identical 2jhp as the external reference. The image shows the interatomic distance in 2jha plotted against the corresponding distance in 2jhp. Distances corresponding to atom pairs separated by one chemical bond are shown in red, those separated by two bonds are shown in blue and all others are shown in black.

The distribution of vectors from the first atom to the second is given by 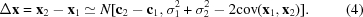
If the atoms are close then their positions are likely to be positively correlated, which will reduce the variability of the distance between them. Conversely, if the atoms are far apart then it is reasonable to surmise that their positions would be more independent and thus the variability of their interatomic distance would be larger. This is supported by Fig. 2 ▶, which demonstrates lower variability for atom pairs that are closer together.

Given that, under assumption of independence of atomic positions, [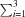 (Δ**x**_*i*_/σ)^2^]^1/2^ follows a noncentral χ distribution with three degrees of freedom with non-centrality parameter {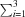
[*E*(Δ**x**_*i*_)/σ]^2^}^1/2^, we deduce that the interatomic distance *D* = [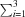
(Δ**x**_*i*_)^2^]^1/2^ is related to the noncentral χ distribution; specifically, *D*σ^−1^ ≃ χ′_3_, where σ^2^ = var(Δ**x**). However, for consistency between *ProSMART* and *REFMAC*5 we use the assumption of a normal distribution of external distances,
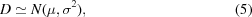
which constitutes the restraint to be used in refinement. Given knowledge of external structural information, we estimate the mean as μ = *r*, the distance between the corresponding atoms in the reference structure. Appropriate selection of the standard deviation σ is less obvious; currently used approaches are described below.

#### 2.3.2. Estimating restraint standard deviations (SDs)

The observed distribution *P*(*d*|*r*) of interatomic distances in the target structure given corresponding distances *r* in the reference structure may be used for selection of restraint SDs. For example, estimation of uniform SDs would allow restraints to be automatically weighted according to the overall agreement between interatomic distances in the two structures. In this trivial case, all SDs are estimated using
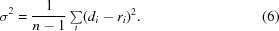
This would result in higher SDs (lower weights) being assigned to all external restraints when the reference structure is less similar to the target. Owing to the distance-dependence of the variability of |*d* − *r*|, using a higher distance threshold *r*_max_ would also result in higher SDs. It follows that the restraints would naturally be weighted down if the target and reference structures exhibit conformational differences.

Another choice would be to allow the SDs to increase with the mean in order to account for the distance-dependence of the observed distribution of restraints. This would allow restraints with small objective values (*r*) to have higher weights, whilst naturally weighting down the longer-range restraints. For example, the restraint variance could be allowed to increase linearly with restraint distance, that is 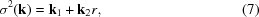
where the parameters **k** = [**k**_1_, **k**_2_] depend on the particular chain pair. This could be justified by the observation that any signalling causing correlation in atomic position would generally become weaker as the restraint distance increases. Furthermore, peculiar behaviour may be observed when there are multiple rigid substructures (*e.g.* domains) present, the effect of which would be exacerbated when the maximum restraint threshold *r*_max_ is large. The presence of multiple domains, or indeed any deterministic conformational changes, would tend to cause a systematic increase in observed restraint error for higher distances *r*.

The maximum-likelihood approach is general enough to allow estimation of parameters from other more complicated functional forms. For example, information such as *B* values or reliability of atomic position if available could be used in the derivation of restraint SDs.

Alternatively, attempts to sensibly estimate restraint SDs may be bypassed, instead allowing the weight of external restraints to be controlled using only the weighting terms in the refinement program.

#### 2.3.3. Maximum-likelihood estimation of parameters

Given a functional form for the restraint variances σ^2^(**k**), we then use maximum-likelihood estimation to optimize the parameters **k**. The optimization problem amounts to searching for parameter values **k** such that the constraints −∂log(*L*)/∂**k** *_j_* = 0 are satisfied for all *j* within some acceptable error margin, so that the likelihood function *L*(**k**) is sufficiently maximized. In general, the probability density function of *D* is given by
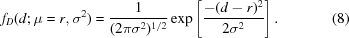
Parameters of SDs are estimated by minimizing the −log likelihood, the gradient of which is given by 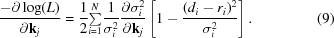
Note that other distributional forms could be considered and handled using this method. Minimization is performed using a quasi-Newton method, in which an approximation of the Hessian matrix is updated after each procedural iteration. Specifically, we use the BFGS formula for updating the inverse Hessian approximation and a line-search algorithm for selecting trial parameter values as described by Nocedal & Wright (1999 ▶).

### 2.4. Fragment-based restraints

Further to using a reference structure, *ProSMART* is able to generate restraints based on individual structural units. This functionality may have broad application, including in the generation of restraints for secondary-structural elements. In particular, external restraints may be generated using an *n*-­residue fragment representing an ‘ideal’ α-helix, which may be used to keep helical structures intact. Such restraints might be used when a suitable reference structure is not available or when the reference chain is itself not sufficiently well refined. However, the suitability of other general in-sequence fragment-based restraints, such as for β-strands, is less obvious owing to their comparatively high degrees of flexibility and the fact that hydrogen bonding occurs between, not within, β-­strands. Another potential application would be when it is desired for a particular region to adopt a known conformation (*e.g.* if a specific small portion of conserved structure is found between the target and a reference chain); the suitability of such an approach would have to be carefully considered for the particular case.

Since aligned fragments may overlap (*e.g.* consecutive helical fragments), it is possible for a particular atom pair to be restrained to several atom pairs in the reference fragment. For example, in a helical fragment the distances between main-chain atoms in residues *i* and *j* may be very similar to those in residues *i* + 1 and *j* + 1. In such cases, restraints for a target atom pair in a helix might be generated using corresponding atoms from residues *i* and *j* or those from residues *i* + 1 and *j* + 1. Consequently, it is necessary to decide which residues to use for restraint generation. More generally, any restraint between atoms from residues *i* and *j* may result from several fragment alignments. Specifically, the reference fragment which has residue range [1, *n*] may be aligned to any of the residue ranges [*j* − *n* + 1, *j*], …, [*i*, *i* + *n* − 1] in the target structure, whilst still implying correspondences for residues *i* and *j* (where *j* − *n* < *i* < *j*). Therefore, ignoring heterogeneities and boundary conditions, there may be up to *i* − *j* + *n* potential alignments of residues *i* and *j* with some residues in the reference fragment.

The list of potential residue correspondences is reduced by fragment score criteria, since we only want to generate fragment-based restraints for regions of structure sufficiently similar to the reference fragment; only configurations with associated Procrustes dissimilarity (local r.m.s.d.) scores below some threshold are included. Of the remaining potential residue-pair alignments, if any, the one with the most favourable associated fragment Procrustes score is selected for restraint generation.

### 2.5. Examples of usage

Here, we present examples of the re-refinement of models previously deposited in the Protein Data Bank (Berman *et al.*, 2002 ▶). Where appropriate, models were re-refined using 30 iterations of refinement by *REFMAC*5 v.5.7 using external structural restraints generated by *ProSMART*; distance-dependent SDs were used for all external restraints. Illustrations of protein structures were generated using *CCP*4*mg* (McNicholas *et al.*, 2011 ▶), with comparative structural analyses performed by *ProSMART*. Model validation (geometry and contact analysis) was performed using the *MolProbity* server (Chen *et al.*, 2010 ▶; Davis *et al.*, 2007 ▶). Graphs were generated using *R* (R Development Core Team, 2011 ▶).

The consideration of altering some major parameters (*w*_ext_, κ, *r*_max_) demonstrates typical behaviour that might be expected in simple cases. Here, parameters were selected by optimizing *R*_free_ by trial and error, although it should be noted that other criteria may be chosen (*e.g.* −LL_free_).

In our examples, refinement is automated, largely using default settings, and no attempt is made to achieve ‘good’ final models. Refinement quality of local regions is not considered given the present purpose, in which we are interested only in the qualitative effect of external restraints on global statistics. Indeed, the examples shown here are neither further improved nor manually inspected following refinement; better models, and thus statistics, would be achieved by optimizing other refinement parameters and by subsequent iterations of manual and automated model building and refinement.

It should be noted that the examples of the re-refinement of deposited models presented here may not represent typical application, since external restraints may also be applied during earlier stages of the refinement process in order to help models to adopt more reliable conformations.

#### 2.5.1. Application of external structural restraints

**Figure 3:**
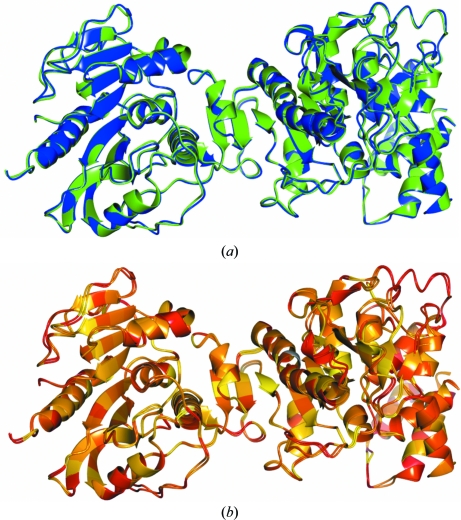
Depictions of superposed models 2jha and 2jhp (*a*) coloured blue (2jha) and green (2jhp) and (*b*) coloured according to structural conservation (r.m.s.d.) of side-chain atoms relative to the residues’ local coordinate frames. Residues with different side-chain conformations are coloured red (*d* > 1 Å), fading through orange (*d* ≃ 0.5 Å) to yellow (*d* ≃ 0 Å) for highly conserved side chains.

Re-refinement of the 3.4 Å resolution structure with PDB code 2jha (Sutton *et al.*, 2007 ▶) was attempted using both main-chain and side-chain external restraints from the sequence-identical 2.5 Å resolution structure 2jhp. Both the target (2jha) and external reference (2jhp) structures comprise one chain crystallized in space group *P*6_5_22. As can be seen in Fig. 3 ▶, they share very similar global conformations. However, the backbone trace is not identical. At a local level, differences in backbone conformation can be detected in a few regions and many residues have different side-chain conformations. For the purpose of this example, it is unknown/unassumed whether these differences are real, *i.e.* a consequence of suboptimal refinement (of target or reference structures), or actual conformational differences. In practice, the reference structure would be manually inspected for poorly built/refined regions of the model.

As can be seen in Table 1 ▶, the unoptimized re-refinement of 2jha without external restraints results in a greatly reduced *R* factor. However, Δ*R* becomes large, suggesting that the re-refined model suffers from overfitting. The use of external restraints from 2jhp results in a considerable decrease in *R*_free_ and −LL_free_ and also a reduced Δ*R*, suggesting that the external structural information stabilizes refinement and increases model reliability in this case. The parameters *w*_ext_ = 7.6, κ = 1.0 and *r*_max_ = 4.2 were chosen so as to minimize *R*_free_. Since refinement statistics alone are not sufficient to unambiguously deduce model improvement, we also consider statistics provided by the *MolProbity* validation server. Refinement with external restraints results in improved geometry and a reduced number of steric clashes, suggesting an improved model, in agreement with our previous assertions based on refinement statistics.

**Figure 4:**
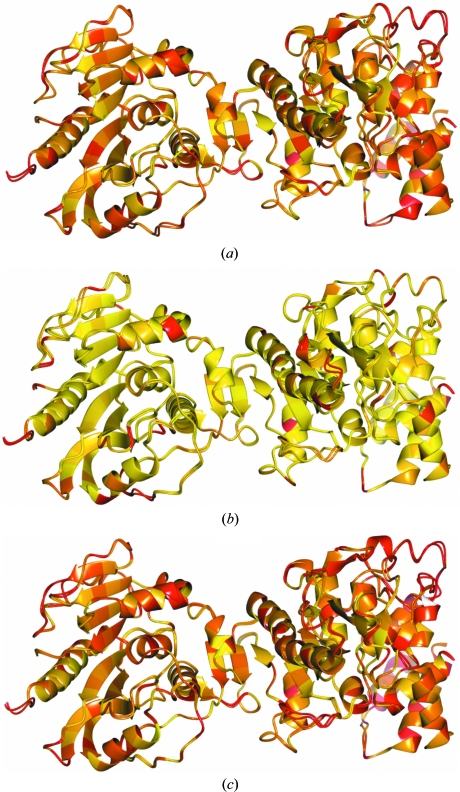
Representations of the main chains of various superposed models coloured by side-chain conformational similarity. Images correspond to a comparison of the model of 2jha after refinement with main-chain and side-chain external restraints from 2jhp (with *w*_ext_ = 7.6, κ = 1.0 and *r*_max_ = 4.2) and (*a*) the original model 2jha, (*b*) the reference model 2jhp and (*c*) the model refined without external restraints. Each image displays the globally superposed compared models, with residues coloured according to structural conservation (r.m.s.d.) of side-chain atoms relative to the residues’ local coordinate frames. Residues with different side-chain conformations are coloured red (*d*> 1 Å), fading through orange (*d* ≃ 0.5 Å) to yellow (*d* ≃ 0 Å) for highly conserved side chains.

In order to understand the influence of external restraints, it is of interest to perform a structural comparison of the re-­refined model with the target and reference models. It is important to confirm that the target structure is not restrained too tightly to the conformation of the reference; regions of structure that are actually different between the target and reference structures should be allowed to differ. In this particular case, the global r.m.s.d. of main-chain atoms between the externally restrained re-refined structure and the target 2jha (and the reference 2jhp) is 0.442 (and 0.375), indicating that the re-refined structure has diverged from the conformation of 2jha whilst not being restrained too tightly to 2jhp. Furthermore, it is possible to see in Fig. 4 ▶ that some regions of backbone structure have remained close to the conformation of the original structure 2jha.

Robust estimation using the Geman–McClure function for the contribution of external restraints to the likelihood function, which reduces the effect of outliers, helps to ensure that regions of structure that correspond to actual differences between the target and reference structures are not restrained too tightly. As illustrated in Fig. 4 ▶, there are multiple residues whose side chains (and also some regions of backbone) have not been pulled into the local conformation of 2jhp (these residues are coloured red). These differences may represent actual differences between the crystals or errors in one of the models; manual inspection of such regions may reveal opportunities for further model improvement.

In contrast, the side chains of many residues have adopted very similar conformations to those in the reference structure (coloured yellow), whilst departing from the conformations of those in the original structure 2jha and the model re-refined without external restraints. This may be reasonable: the data corresponding to the target structure may be so poor that the bias introduced by the reference model appropriately increases model reliability (as in this example). In other cases where the restraints are deemed too tight it may be appropriate to alter relevant parameters, exclude certain residues from having external restraints or restrain only main-chain atoms.

It is important to acknowledge that the selection of certain parameters, most notably the external restraints weight *w*_ext_, the Geman–McClure parameter κ and the maximum restraint length *r*_max_, can have a very large effect on refinement. Furthermore, the appropriate choice of parameter values seems to be different for different cases, meaning that trial and error is currently required in order to produce reasonable results when using external structural restraints. Unfortunately, these parameters are highly correlated. The appropriate choice of parameters may depend on various factors such as data quality, resolution, other refinement parameters, the suitability of the external reference structure and whether or not side-chain atoms are to be restrained. Consequently, careful consideration should be made in the application of external restraints.

**Figure 5:**
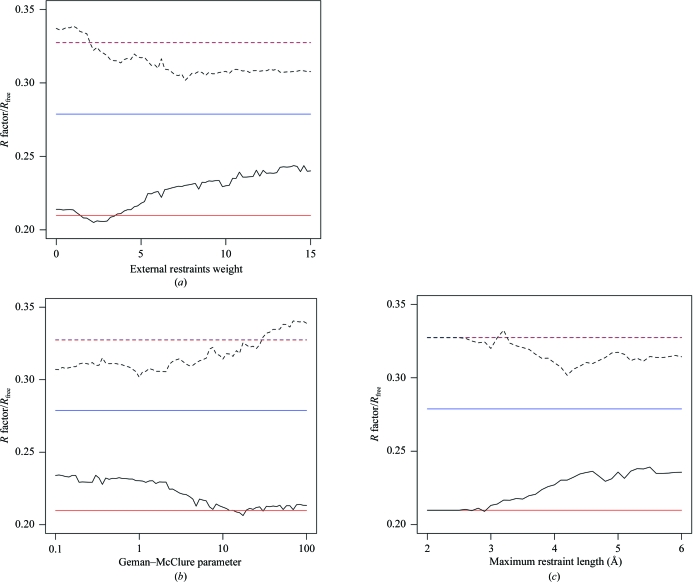
*R* factor (solid lines) and *R*_free_ (dashed lines) after 30 *REFMAC*5 refinement iterations starting from the model 2jha plotted against (*a*) the external restraints weight *w*_ext_, (*b*) the Geman–McClure weight κ on a logarithmic scale and (*c*) the maximum external restraint length *r*_max_. Lines correspond to the original model (blue), the model refined without external restraints (red) and the model refined with external restraints from the reference structure 2jhp (black), generating restraints for both main-chain and side-chain atoms. In each graph, the two parameters not being considered were fixed to the values that globally minimized *R*_free_, *i.e.* *w*_ext_ = 7.6, κ = 1.0, *r*_max_ = 4.2.

Fig. 5 ▶ shows the range of refinement statistics achieved using different values of the parameters *w*_ext_, κ and *r*_max_, demonstrating that the choice of these parameters is important for the successful application of external restraints.

When the influence of external restraints is weak (small *w*_ext_ or large κ) the external restraints have little or no positive effect on refinement. If the external restraints do not contain sufficient positive information then the refinement statistics may worsen (*R* and *R*_free_ will rise). This negative effect can be countered by introducing restraints that have a high useful information content (requiring a suitable choice of reference structure), as is observed for intermediate parameter values in this case.

Conversely, selecting very high weights (high *w*_ext_ or low κ) has the effect of restraining the model too tightly to the reference structure, with the restraints behaving more like constraints. This often results in greatly increased values of *R* and *R*_free_ depending on the structural similarity of the target and reference models.

Refinement statistics arising from a variety of maximum restraint lengths *r*_max_ are shown in Fig. 5 ▶(*c*). Using a low *r*_max_ results in relatively few restraints being generated, thus having little effect on refinement. As *r*_max_ increases more restraints are generated and the external restraints have a greater impact on refinement. Note that longer restraints are less tolerant of conformational change, influencing tighter globally rigid structural agreement with the reference structure. Therefore, using larger values of *r*_max_ has strong negative effects in cases where conformational changes are present between target and reference structures. However, the effect of this is not dramatic in this example, since the structures are well conserved at the global level.

#### 2.5.2. Fragment-based α-helix restraints

We now consider the re-refinement of the 3.3 Å resolution model of human haemoglobin with PDB code 1ydz (Kavanaugh *et al.*, 2005 ▶). The use of external restraints is demonstrated using fragment restraints from an ideal helix (using a fragment length of five residues and a Procrustes dissimilarity score threshold of 0.3 Å) and also external restraints from a near-sequence-identical 1.07 Å resolution reference structure 2w72 (Savino *et al.*, 2009 ▶). Both the target (1ydz; space group *P*2_1_2_1_2) and the reference (2w72; space group *P*2_1_) structures comprise four sub­units.

As can be seen in Table 2 ▶, re-refinement of 1ydz without external restraints results in improved refinement statistics, particularly when using local NCS restraints. Application of helix restraints results in a further decrease in *R*_free_ and Δ*R* (although an increased −LL_free_; this may or may not have been the case were −LL_free_ used as the optimization criteria). More substantial improvements in refinement statistics are achieved by using external restraints from 2w72 both when using only main-chain restraints and when using both main-chain and side-chain restraints. Such situations, where the use of external structural information from both main-chain and side-chain atoms appears to improve the model, arise owing to local relative atomic positions being highly conserved between the reference and (actual) target structures. Thus, in such cases information contained in the reference structure can successfully be used to improve the model of the low-resolution structure. However, in other cases where the reference structure is less similar to the target the use of external side-chain restraints may not be appropriate.

It may be argued that both the homologous structures and the α-helical fragment appear to provide useful information that has a positive effect on crystallographic refinement in this case. These assertions are also supported by general improvements in *MolProbity* validation scores, although one would anticipate subsequent manual refinement to result in substantial further improvements.

Evidence suggests that external restraints on the homologous structure have a greater positive impact than restraints on the presumed helical conformation. This makes sense, since the helical restraints generically pull local backbone structure towards the α-helix attractor, whilst external restraints from 2w72 contain information specific to the particular protein class. The use of a library of helical fragments may result in further improvements. We conclude that helix restraints may be useful in some cases, particularly when appropriate high-resolution reference structures are not available. More generally, given an appropriate fragment library, it may be possible to generate restraints to attractors in fragment-conformation space using this method.

**Figure 6:**
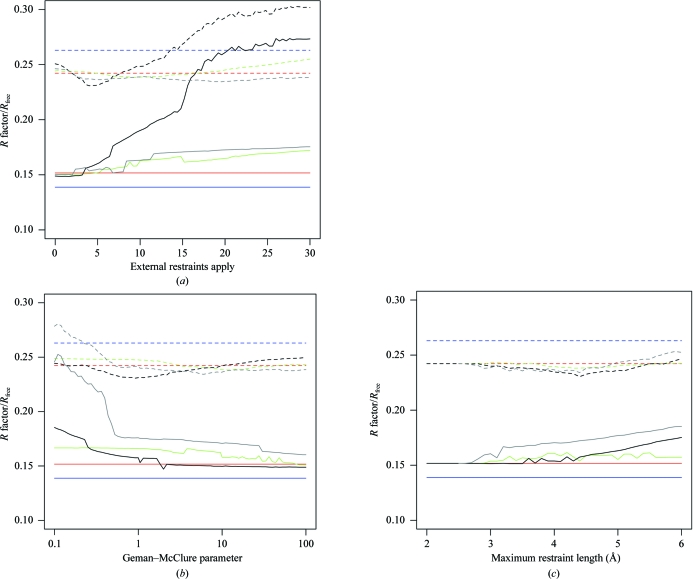
*R* factor (solid lines) and *R*_free_ (dashed lines) after 40 *REFMAC*5 refinement iterations starting from the model 1ydz plotted against (*a*) the external restraints weight *w*_ext_, (*b*) the Geman–McClure weight κ on a logarithmic scale and (*c*) the maximum external restraint length *r*_max_. Lines correspond to the original model (blue), the model refined with local NCS restraints but without external restraints (red) and the model refined with local NCS restraints and α-helical restraints (green), external main-chain restraints from 2w72 (grey) and external main-chain and side-chain restraints from 2w72 (black). In each graph, the two parameters not being considered were fixed to the values that globally minimized *R*_free_, *i.e.* *w*_ext_, κ and *r*_max_ are 8.9, 13 and 4.3, respectively, for α-helical restraints, 19, 5.9 and 4.4, respectively, for main-chain restraints and 4.2, 0.94 and 4.4, respectively, for main-chain and side-chain restraints.

Fig. 6 ▶ demonstrates the effect of altering the external restraint parameters *w*_ext_, κ and *r*_max_ from their optimal values (according to *R*_free_ minimization criteria). These parameters are found to be optimal at different values depending on whether helical restraints, main-chain restraints or main-chain and side-chain restraints are applied. We conclude that the naive application of external restraints without parameter optimization can result in reduced model quality, even in cases where the reference structure is an appropriate choice. The appropriate choice of parameters may be very different in different cases. The successful application of external restraints requires the suitable selection of reference structure(s), atom pairs, estimation of SDs and refinement parameters.

## 3. Map sharpening

The map-sharpening problem can be written in the general form 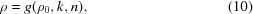
where ρ_0_ is the underlying signal that we would like to observe (actual electron density), ρ is the observed signal (model of electron density from observation), *g* is a process through which blurring operates on the signal, *k* is a blurring function that changes the signal (ρ_0_) before observation is carried out and *n* is noise. However, this formulation is too general to be practical. In order to make the problem manageable, we must make assumptions regarding the functional forms of *g* and *k* and assume a model for the noise *n*. Therefore, for simplicity, we assume that noise is additive and the blurring function is linear,
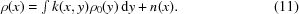
If there were no noise then the problem would be a linear equation. This problem is ill-posed, especially when *k* is near singular, *i.e.* small perturbations in input parameters may cause large variations in output. For example, the effects of small noise addition, an incorrectly defined blurring function or Fourier series termination may result in an uninterpretable ‘deblurred’ electron-density map. It should be noted that in crystallography we always deal with limited noisy data and that Fourier series termination is always present. Even if there were no noise and we had knowledge of the exact blurring function *k*(*x, y*), solving (11) would still not be straightforward. The numbers of equations and parameters to be estimated are equal to the number of grid points in the electron density, which can be very large.

The problem becomes manageable, whilst not completely reflecting reality, when we make the further assumption that the blurring function is independent of position. This simplification essentially means that the whole content of the asymmetric unit oscillates as a unit with no rotational component, resulting in the blurring function having the property *k*(*x, y*) = *k*(*x* − *y*, 0). Using the notation *k*(*x*) = *k*(*x*, 0), (11) becomes
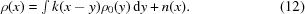
Since the problem is ill-posed, we can approach its solution utilizing ideas from the field of regularization (Tikhonov & Arsenin, 1977 ▶). Under the assumption of white noise, our ill-posed problem may be replaced by the minimization problem 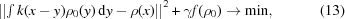
where ||.|| denotes the *L*_2_ norm, *f* is a regularization function and γ is a regularization parameter to be selected. Usually, regularizers are chosen so that the resultant function obeys certain conditions. For example purposes, we shall consider two popular conditions: (i) the function should be small and (ii) the first derivatives of the function should be small (*i.e.* the function should vary slowly). For the first case we have 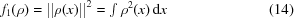
and for the second case
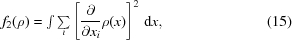
which is known as a first-order Sobolev norm. Since ρ is a periodic function, we can write
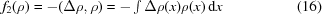
where Δ is the Laplace operator [Δ = 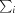
(∂^2^/∂*x_i_* ^2^)] and (.,.) denotes the scalar product in Hilbert space.

Now the problem is reduced to finding the minimum of the functional
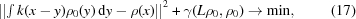
where *L* = *I* (identity operator) for *L*_2_-type regularizers (first case) and *L* = −Δ for Sobolev-type regularizers (second case).

Using Plancherel’s theorem, the convolution theorem and the fact that the Fourier transformation of the Laplacian is proportional to the negative squared length of the reciprocal-space vector, we can rewrite the problem as
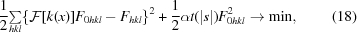
where *F*_*hkl*_ is the structure factor before sharpening (*e.g.* 2*mF*_o_ − *DF*_c_-type maps), *F*_0*hkl*_ is that after sharpening and |*s*| = 2sinθ/λ is the length of the reciprocal-space vector, with *t*(*s*) = 1, α = γ for regularization function *f*_1_ and *t*(*s*) = *s* ^2^, α = (2π)^2^γ for *f*_2_. This minimization problem has a very simple solution,
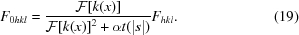
When *k*(*x*) is Gaussian then the equation has an especially simple form, since *K*(*s*) = 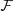
[*k*(*x*)] = exp(−*s^T^B*_deblur_ *s*/4), where *B*_deblur_ is an anisotropic deblurring *B* value.

Unfortunately, in reality neither *B* values nor α are known. Whilst there are several techniques to find an ‘optimal’ value for α (Vogel, 2002 ▶) when the blurring function is known, in our implementation such an approach did not give consistent results. Therefore, we used the following *ad hoc* procedure for selection of the regularization parameter. Denoting *K*_α_(*s*) = *K*(*s*)/[*K* ^2^(*s*) + α*t*(|*s*|)] and *A*_α_(*s*) = *K*_α_(*s*)*K*(*s*), we see that *A*_α_ is similar to the hat function used in regression analysis (Stuart *et al.*, 2009 ▶). We can define the degrees of freedom of errors (the number of observations minus the effective number of parameters) as^1^

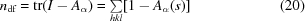
Note that when α = 0 then *n*_df_ = 0 and when α → ∞ then *n*_df_ is equal to the number of observations. We select α so that *n*_df_ is equal to 10–20% of the number of observations. Since we do not know the exact values of *B* and α, we also perform *ad hoc* integration using an empirically derived distribution of these parameters. The necessary integral may then be written
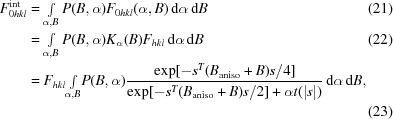
where *B*_aniso_ reflects the anisotropy of the data and is calculated during scaling of the calculated structure factors relative to the observed structure factors, under the conditions that it obeys crystal symmetry, and tr(*B*_aniso_) = 0.

The joint probability distribution of *B* and α can be written 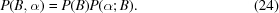
The mean value of the isotropic part is taken to be equal to the median value (*B*) of the coordinates (although it may be better to use Wilson’s *B* value estimated using intensity curves derived by Popov & Bourenkov (2003 ▶). We approximate the distribution of the isotropic part of the *B* values using a Gaussian distribution centred at *B*_sharp_ with standard deviation equal to *B*_sharp_/10. For each *B* value, we select α so that *n*_df_ is 10–20% of the number of observations and the standard deviation of the distribution of α is taken to be α_*B*_/10.

Note that (17) and (18) suggest a class of regularizers. They can be selected to use particular knowledge about the electron density in real and reciprocal space. For example, if it is desired to suppress the effect of ice rings then one can select *t*(|*s*|) so that the corresponding reflections are weighted down.

### 3.1. Implementations and an example

We have implemented anisotropic sharpening with *L*_2_ and Tikhonov–Sobolev regularizers with and without integration over the *ad hoc* joint probability distribution of *B* and α using probability distribution (24). We have also implemented the regularization function *t*(*s*) = 1 + *s* ^2^. These are available from *REFMAC*5 v.5.7. In our tests, all regularization functions gave similar results. This is not surprising, as the major problem is that the blurring function is not position-independent. Before finding accurate regularizers, the problem of modelling position-dependent blurring functions should be dealt with. All results presented here were achieved using the *L*_2_-type regularizer.

**Figure 7:**
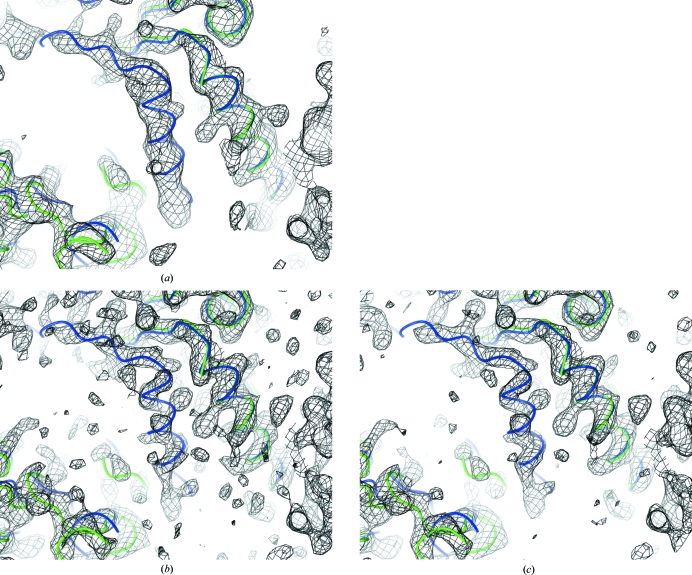
Visual effects of map sharpening on electron density. This example was taken from PDB entry 2r6c. Images show the map with (*a*) no map sharpening, (*b*) map sharpening using the inverse filter (no regularization) and (*c*) a regularized sharpened map using the *L*_2_-type Tikhonov regularizer, with sharpening coefficients integrated over *B* and α, as described in the text. The backbone trace of 2r6c chain *C* is shown in green. The homologous structure 2r6a chain *A* is shown in blue, superposed using residues 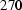
–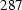 from 2r6a chain *A*. The image shows unmodelled density in 2r6c that corresponds to a helix present in 2r6a. Both sharpened maps show more features than the unsharpened map, with the regularized map giving more connectivity. Images were generated using *CCP*4*mg* (McNicholas *et al.*, 2011 ▶).

Map sharpening was tested for many cases using data sets from the PDB (Berman *et al.*, 2002 ▶) with resolution below 3 Å. The best results were obtained for PDB entry 2r6c (Bailey *et al.*, 2007 ▶). For any low-resolution data taken from the PDB, before map calculation we generally try jelly-body, local NCS (if present) and external reference structure (if applicable) restrained refinement and take the best refined results for further analysis. For 2r6c, the original *R*/*R*_free_ statistics reported in the PDB were 0.321/0.344. After refinement, these values became 0.240/0.300. Fig. 7 ▶ shows an illustration of the maps after refinement with and without unregularized and regularized map sharpening. It is apparent that in this case using regularized map-sharpening coefficients shows more features (possibly side chains) and connectivity. Whilst this example shows regularization using the *L*_2_-type regularizer, it should be noted that the Sobolev-type regularizer gave similar results.

## 4. Conclusions and future directions

In this paper, we have presented two tools to aid in low-resolution refinement, namely external structural restraints and regularized map sharpening.

The use of external restraints based on homologous reference structures and/or structural fragments gives promising results. In particular, we have demonstrated how improved models can be achieved by the externally restrained re-refinement of deposited models.

Since the use of external restraints will alter global geometry validation statistics, such results should be interpreted accordingly and the integrity of local structure should always be considered. Indeed, local regions should still be manually inspected in order to ensure local suitability of the use of external restraints, despite any apparent improvements in overall statistics. If there are any serious artefacts that arise owing to bias towards the reference structure, it may be appropriate to exclude particular residues from external restraint generation.

In some cases, better results can be achieved by utilizing information from multiple reference structures, the difficulty often being that this requires the existence and availability of multiple structures suitable as references. Our implementation allows the generation of external restraints based on multiple reference structures; currently, the restraints most consistent with the target model are selected for use during refinement.

For practical application, we anticipate external restraints to also be of particular use during intermediate stages of model building/refinement, for stabilizing local structure and in helping to achieve sensible model geometry. Of course, the degree of any improvement owing to external restraints will be limited by the quality of the reference structure. For example, the *MolProbity* statistics for the reference structure 2jhp (used in one of our examples) are not particularly low given its resolution (see Table 1 ▶). Nevertheless, structural information contained in this reference model was able to improve the lower resolution target. However, the use of a more reliable reference model may have resulted in further improvements to the target structure 2jha. This scenario highlights the immediate need for ways to automatically validate the suitability of reference structures, most importantly at the local level, so that destructive restraints are not generated or are appropriately weighted down (whilst down-weighting is already effectively performed by using robust estimators in our implementation, other complementary approaches would be desirable). In application, it may be sensible to attempt re-­refinement of any reference structures before restraint generation in an attempt to improve the quality of the prior information. For example, this might be achieved automatically by using the *PDB_REDO* protocol (Joosten *et al.*, 2009 ▶). In some cases, manual model rebuilding and refinement of reference structures may be necessary/appropriate and thus should ideally always be considered. Such approaches may reduce any error propagation from reference to target models.

There is much room for improvement and future exploration in the generation and application of external structural information.

(i) The establishment of a method for determining sensible parameter values, most importantly the weight of external restraints *w*_ext_ and also the maximum restraint length *r*_max_. Appropriate choices are unclear at this stage and may depend on various factors, such as the X-ray weight *w*, the quality and resolution of the structure, the number of chains and whether local NCS restraints are used, and on the net local similarity between the target and reference structures.
(ii) The use of non-normal residuals during refinement. For example, the noncentral χ distribution could be used for sufficiently long-range restraints (under the assumption of independence).
(iii) Consideration of the use of different functional forms for estimating restraint SDs. For example, the ability to modify SDs using *B* factors and local structural dissimilarity scores has been implemented, but the suitability of such approaches should be carefully assessed.
(iv) Investigation of the utility of estimating individual restraint distributions for different types/classes of interatomic distances (*e.g.* atom type, bond separation *etc*.). Specifically, it may be of benefit to further explore the effect of bond separation on restraint variability and the subsequent effect on crystallographic refinement.
(v) Expansion of the approach of restraint generation and SD estimation to better utilize situations where multiple reference structures are available. In the current implementation all restraints are pooled or alternatively only the ‘best’ restraints are selected. A more sophisticated solution would be to more appropriately describe the distribution of each interatomic distance restraint. This would result in the assignment of bespoke restraints for each individual atom pair that more closely represent reality, being based on observed intraclass flexibility. However, this would require an appropriate array of reference structures, which may include different forms/models of the same protein, classes of structurally similar proteins and structure ensembles resulting from other experimental (NMR) or theoretical (MD) techniques.
(vi) Accounting for errors in reference structures, ensuring that such errors are not transferred to the target structure. Such errors may be identified independently (*e.g.* local geometry validation) or by the assessment of local structural similarity of target and reference chains (although it would be unclear whether such dissimilarities would be a consequence of actual differences, errors in the target structure or errors in the reference structure).
(vii) Consideration of generic restraints derived from considering the density of fragment-conformation space. This may allow the expansion and generalization of the presented fragment-based approach into an automated method, which is currently only recommended for α-helical restraints and for cases afforded special manual consideration.
(viii) Assessment and identification of structures appropriate for use as external references, given a target. Currently, reference structures are manually identified and suitability is manually assessed. It would be desirable for such decisions to be reliably automated, *e.g.* using *BALBES* (Long *et al.*, 2008 ▶). A related problem is the automatic removal or weighting down of restraints from regions of poor quality in the reference models.
(ix) Multicrystal refinement, whereby multiple data sets are used to achieve a single model (as in multicrystal averaging). Each model would be a refinement target, as well as being used as a reference structure for all other models. Successful implementation of this is an important future prospect for low-resolution refinement.

We have also implemented DNA/RNA base-pair restraints based on interatomic distances, torsion angles and chirality; testing is currently in progress. Parameters for these restraints have been taken from Neidle (2008 ▶). For accurate refinement of DNA/RNA it is necessary to use sugar-puckering as well as base-stacking restraints. Whilst it is relatively simple to implement sugar-puckering restraints, *e.g.* using the elegant method presented by Cremer & Pople (1975 ▶), determining appropriate distributional parameters will take some time. Designing restraints for base stacking is a much more challenging problem, for which we do not currently have any satisfactory approaches.

The implemented method of regularized map sharpening uses the assumption that the blurring function is position-independent. However, this assumption may not always be valid: it is expected that the oscillation of molecules within a crystal will be more complex and crystal disorder will be more anisotropic. One natural extension to map sharpening would be to use TLS parameters (Winn *et al.*, 2001 ▶) as a blurring function. However, we are not aware of a simple solution to this problem. Future work will include deblurring using TLS parameters. Another problem with the current approach is that we assume that noise and signal are uncorrelated and that the noise is white noise. This may not reflect reality, especially when atomic model errors are dominating contributors to the noise. For density modification, the problem may become even more complicated.

## Figures and Tables

**Table 1:** Model-refinement statistics *R* factor, *R*_free_, Δ*R* = *R*_free_ − *R* and −LL_free_, and ‘clashscore’ and ‘*MolProbity* score’ from model validation using *MolProbity* Percentages in parentheses indicate how the score compares amongst structures of comparable resolution (larger is better). Refinement statistics were calculated using *REFMAC*5 (original *R*/*R*_free_ values in the PDB files were 0.240/0.331 for 2jha and 0.226/0.289 for 2jhp). Note that (unnormalized) values of −LL_free_ are not comparable for different structures and are quoted for the model achieved by optimizing *R*_free_ (when using external restraints).

Model	*R*	*R*_free_	Δ*R*	−LL_free_	Clashscore	*MolProbity* score
Original (2jha)	0.2788	0.3275	0.0487	3276	49.05 (56%)	3.85 (37%)
Reference (2jhp)	0.3107	0.3504	0.0397	8294	23.06 (60%)	3.06 (33%)
Refined without external restraints	0.2098	0.3273	0.1175	3292	50.04 (54%)	3.81 (41%)
Refined with main/side-chain restraints	0.2303	0.3017	0.0714	3197	23.69 (89%)	3.09 (83%)

**Table 2:** Model-refinement statistics *R* factor, *R*_free_, Δ*R* = *R*_free_ − *R* and −LL_free_, and ‘clashscore’ and ‘*MolProbity* score’ from model validation using *MolProbity* Percentages in parentheses indicate how the score compares amongst structures of comparable resolution (larger is better). Refinement statistics were calculated using *REFMAC5* (original *R*/*R*_free_ values in the PDB files were 0.127/0.307 for 1ydz and 0.129/0.153 for 2w72). Note that (unnormalized) values of −LL_free_ are not comparable for different structures and are quoted for the model achieved by optimizing *R*_free_ (when using external restraints).

Model	*R*	*R*_free_	Δ*R*	−LL_free_	Clashscore	*MolProbity* score
Original (1ydz)	0.1388	0.2630	0.1242	5389	39.02 (67%)	3.20 (77%)
Reference (2w72)	0.2395	0.2465	0.0070	53516	11.54 (19%)	1.80 (35%)
Refined without external restraints	0.1391	0.2594	0.1203	5367	28.41 (86%)	3.08 (82%)
Refined with local NCSR without external restraints	0.1517	0.2422	0.0905	5287	21.22 (91%)	2.74 (94%)
Local NCSR and α-helix restraints	0.1576	0.2381	0.0805	5300	21.00 (91%)	2.68 (96%)
Local NCSR and external main-chain restraints	0.1722	0.2342	0.0620	5271	15.81 (97%)	2.44 (98%)
Local NCSR and external main/side-chain restraints	0.1574	0.2304	0.0730	5245	16.69 (97%)	2.45 (98%)

## Footnotes

1 This is the simplest way of defining effective degrees of freedom of errors. Another formula is *n*_df_ = tr(*I* − *A*_α_)^2^. Equation (20) has a simple form in reciprocal space, when blurring is position-independent then the sharpening matrices are diagonal.
